# Education level and physical functional limitations among Japanese community residents-gender difference in prognosis from stroke

**DOI:** 10.1186/1471-2458-9-131

**Published:** 2009-05-09

**Authors:** Kaori Honjo, Hiroyasu Iso, Ai Ikeda, Manami Inoue, Shoichiro Tsugane, The JPHC Study Group

**Affiliations:** 1 Department of Social and Environmental Health, Public Health, Osaka University Graduate School of Medicine, Osaka, Japan; 2 Epidemiology and Prevention Division, Research Center for Cancer Prevention and Screening, National Cancer Center, Tokyo, Japan

## Abstract

**Background:**

Little research has been conducted to examine the relationship between education level and functional limitations among Japanese community residents. We sought to examine the association between education level and physical functional limitations among Japanese men and women, and whether that association was modified by gender and history of stroke.

**Methods:**

We examined prevalence of physical functional limitation by educational level using the data from a total of 29,134 Japanese men and women aged 50–69 years living in communities in 2000. The information of educational level (junior high school graduates, senior high school graduates, college and/or higher education) and physical functional limitations (no need for assistance, need for assistance when going outdoors, and need for assistance to carry out indoor activities) were obtained by self-administrated questionnaire.

**Results:**

The proportions of the subjects reported their highest level of schooling were 48% for junior high school, 39% for high school, and 13% for college. Three hundred and twenty eight subjects (1% of total subjects) reported having some physical functional limitations. Multinomial logistic regression analyses showed that the odds ratio of needing assistance to carry out indoor activities were 4.84(95%CI:3.61,6.50) for lowest education level group and 2.21(95%CI:1.00,4.86) for middle education level group compared to highest education level group. The corresponding odds ratios of needing assistance when going outdoors were 2.36(95%CI: 2.03,2.72) and 1.08(95%CI:0.73,1.60), respectively. Further, the significant excess prevalence of having functional limitations associated with the low education level was identified for men regardless of history of stroke and for women without history of stroke.

**Conclusion:**

Low education level was associated with the higher prevalence of physical functional limitations for both genders. That association among persons with history of stroke was observed for men but not for women probably due to gender differences in stroke subtypes and social support.

## Background

Japan is one of the most rapidly aging countries in the world [1]; the number of elderly who were certified as being in need of care has been rapidly increasing since the governmental long-tem care insurance system was introduced in 2000 [2]. Recent epidemiological studies in Europe and the United States reported that lower education level was associated with poorer functional limitation level among elderly [3,4]. In Japan, however, few studies have been conducted to examine the relationship between education level and functional limitations.

Educational attainment is a strong factor in the formation of social consciousness regarding social stratification in Japan [5,6], and differences in educational levels contribute to health inequalities [7-9]. Low educational levels has been associated with cognitive impairment [10], unhealthy behaviors [9], and history of falling [11], which lead a hypothesis that unequal prevalence of physical functional limitation by education level could exist in Japan.

Stroke is one of the major reasons for functional limitation among elderly in Japan; 30% of people who have functional limitations reported an onset of stroke as a main reason for functional limitation [12]. Based on this fact, it is hypothesized that different risk of stroke incidence by education level could be one of the possible mechanisms for unequal prevalence of functional limitations by education level. There is some evidence to suggest that people with low socioeconomic status were likely to develop severer stroke [13,14], to have dependency for activities of daily living at 28 days after stroke's onset [15] and longer-term disability [14,15], compared to those with high socioeconomic status.

A recent study also showed that low socioeconomic status led limited access to therapeutic measures of acute stroke care [16]. Limited access to rehabilitation and material and psychosocial resources were suggested as possible mechanisms for unequal distribution of functional limitation after stroke [13]. Therefore, we hypothesized that the association between education level and functional limitations may also exist among stroke patients. As far as we know, no study has been conducted to examine the association between education level and prevalence of functional limitations, paying attention to prognosis after stroke in Japan.

The aim of this study was to conduct a cross-sectional examination of the associations between education level and functional limitations among Japanese community residents. The hypotheses for this study were 1) men and women with low educational attainment would be more likely to have physical functional limitations compared to those with high educational attainment, and 2) those associations would be observed regardless of history of stroke. This study will have implications for how the educational background is related to inequalities in the physical limitations arising as a result of stroke. For example, health education programs focusing on people with low education level would reduce gap in incidence of functional limitation by education level, while policies assuring equal access to resources for medical treatment, rehabilitation, and psychosocial supports would reduce the inequalities in prognosis.

## Methods

### Subjects

We used the data from the Japan Public Health Center-based Prospective (JPHC) Study consisting of 54,498 participants registered in 14 administrative districts on January 1, 1990. The sampling design and procedures have been described in detail elsewhere[17]. This study was approved by the human ethics review committees of the National Cancer Center.

The baseline self-administered questionnaire was distributed to 54,498 registered non-institutionalized community residents who were 50–69 in 2000, and 43,149 participants returned their questionnaire with a response rate of 79%. A self-administered follow-up survey was conducted for all baseline participants in 2000. Eventually, 36,213 participants completed follow-up survey for a response rate of 84%.

This study focused on the participants at the follow-up survey who provided data of physical functional limitations. We used the data on educational attainment levels at the baseline survey which would be unlikely to be changed at the follow-up survey. Thus, this study is considered as a cross-sectional study. The number of subjects for this study was 29,134.

### Measurements

#### Physical functional limitations

Physical functional limitation was measured by the self-administered questionnaire in 2000 as an ordinal variable (Table 1). The physical functional limitation scale was divided into three levels: no need for assistance (0–2), need for assistance when going outdoors (3–4), and need for assistance to carry out indoor activities (5–8). We further created a binary outcome, having physical functional limitations, by combining needing assistance when going outdoors and need for assistance to carry out indoor activities.

**Table 1:** Physical Functional Limitation Measurement Scale

**No need for assistance**
0: No physical functional limitations
1: Go outdoors without assistance, using public transportation with some physical functional limitations
2: Go outdoors without assistance within the neighborhood with some physical functional limitations

**Need assistance when going outdoors but able to carry out indoor daily living activities indoors **without assistance
3: Get out of bed completely without assistance and go outdoors sometimes with assistance
4: Stay in bed sometimes and go outdoors less frequently

**Need assistance to carry out most of daily living activities**
5: Get into wheelchair without assistance, and eat meals and use the toilet out of bed.
6: Need assistance to get into wheelchair

**Bed-bound and eat meals, change clothes, and use the toilet with assistance**
7: Can roll over without assistance
8: Cannot roll over without assistance

#### Education level

Education level was measured by self-administered questionnaire in 1990. The subjects reported their age of education completed, which were categorized into three groups: age 14 and younger (0), age 15 to 17 (1), and age 18 and older (2).

#### History of stroke

History of stroke was determined from responses for the self-administered questionnaire in 2000 as well as from surveillance data on stroke incidence. The surveillance procedures have been described in detail elsewhere [18]. We considered the subjects who reported to have history of stroke on the questionnaire and/or those who were confirmed to have suffered stroke by the surveillance data on stroke incidence as subjects with history of stroke.

### Statistical analyses

We used multinomial logistic regression analyses with a generalized logit model to calculate the age-and gender-adjusted odds ratios (ORs) and 95%confidence intervals (CI) for 3-level physical functional limitations by education level [19]. We further conducted stratified analysis by age groups.

We performed ordinal logistic regression analyses stratified by history of stroke by combining the highest and the middle educational groups in order to improve statistical stability. We calculated the age-adjusted ORs (95% CI) for having physical functional limitations according to education level depending on history of stroke. In addition, we further stratified it by gender.

## Results

The selected characteristics of the study population, consisting of adults (50–69 years of age in 2000) who provided with complete information on age, gender, educational attainment, and physical functional limitations (n = 29,134) are presented in Table 2. The proportions of the subjects reported the age of education completed were 48% for age of 14 and younger, 39% for age of 15 to 17, and 13% for age of 18 and older. The proportions of the subjects who reported having some physical functional limitations (1.1%), needing assistance to go outdoors (0.8%), and needing assistance to carry out daily living activities indoors (0.3%). Approximately half (47%) of the subjects were men and their mean and median age was 59 years, the majority (77%) were married, and the proportion of the subjects who reported having a history of stroke was 3%.

**Table 2:** Selected demographic characteristics of study participants, by gender, Japanese Public Health Center Cohort study 1.

	*Male (n = 13,778)*				*Female (n = 15,356)*			
		
N (%)	14 and younger 6,154 (44.7)	15 to 17 5,702 (41.4)	18 and older 1,922 (14.0)	p-value	14 and younger 7,947 (51.8)	15 to 17 5,694 (37.1)	18 and older 1,715 (11.2)	p-value
**Physical functional limitation level**.								
Need assistance when going outdoors	68 (1.1)	28 (0.5)	8 (0.4)	<.0001	92 (1.2)	23 (0.4)	10 (0.6)	<.0001
Need assistance to carry out daily living activities indoors	40 (0.7)	18 (0.3)	2 (0.1)		31 (0.4)	7 (0.1)	1 (0.1)	
								
**Age group**								
50–59	2,529 (41.1)	3,252 (57.0)	1,190 (61.9)	<.0001	3,185 (40.1)	3,287 (57.7)	1,158 (67.5)	<.0001
60–69	3,625 (58.9)	2,450 (43.0)	732 (38.1)		4,762 (59.9)	2,407 (42.3)	557 (32.5)	
								
**Marital status**								
Never married/divorced/separated	1,272 (20.7)	747 (13.1)	222 (11.6)	<.0001	2,766 (34.8)	1,283 (22.5)	390 (22.7)	<.0001
								
**History of stroke**								
Yes	260 (4.2)	197 (3.5)	53 (2.8)	0.005	179 (2.3)	75 (1.3)	29 (1.7)	0.0003
								
**Smoking**								
Never smoker	1,913 (31.2)	1,671 (29.4)	592 (30.9)	<.0001	7,471 (94.8)	5,398 (95.2)	1,603 (94.1)	0.09
Past smoker	1,709 (27.8)	1,647 (29.0)	625 (32.6)		34 (2.0)	68 (1.2)	34 (2.0)	
Current smoker	2,516 (41.0)	2,364 (41.6)	699 (36.5)		308 (3.9)	201 (3.6)	66 (3.9)	
								
**Physical activities**								
Almost none	2,108 (49.3)	1,787 (39.3)	628 (38.1)	<.0001	2,480 (46.9)	1,708 (41.0)	567 (41.1)	<.0001
1–3 times/month)	893 (20.9)	1,334 (29.4)	516 (31.3		887 (16.8)	817 (19.6)	286 (20.7)	
More than once/week	1,271(29.8)	1,423 (31.3)	506 (30.7)		1,925 (36.4)	1,641 (39.4)	527 (38.2)	
								
**Alcohol intake**								
Drinker	4,468 (73.0)	4,556 (80.2)	1,533 (80.0)	<.0001	1,450 (18.4)	1,555 (27.4)	521 (30.7)	<.0001
Quitter	472 (7.7)	106 (5.5)	287 (5.1)		107 (1.4)	54 (1.0)	28 (1.7)	
Non drinker	1,182 (19.3)	838 (14.8)	277 (14.5)		6,314 (80.2)	4,057 (71.6)	1,151 (67.7)	

Older age, non-married status, and history of stroke were inversely associated with educational level for both men and women, while alcohol intake was positively associated with education level. For men, smoking status was associated with education level; the prevalence of current smoker was higher among men with lowest education level compared to those with higher education level (Table 2).

For both genders, education level and alcohol intake were inversely associated with physical functional limitations, while age, non-married status, and history of stroke were positively associated with them. For men, current smoking was inversely associated with physical functional limitations (data not shown).

Table 3 presented the results of multinomial logistic regression analyses, showing the relationship between education level and physical functional limitation. The odds ratios (ORs) (95% confidence intervals (CI)) for physical functional limitations were adjusted for age and gender compared to no need for assistance. The odds ratio for needing assistance to carry out indoor activities was 4.84 (95%CI: 3.61, 6.50) for the lowest educational group and 2.21 (95%CI: 1.00, 4.86) for the middle educational group relative to the highest educational group. The corresponding odds ratios for needing assistance to go out were 2.35 (95%CI: 2.03, 2.72) and 1.08 (95%CI: 0.73, 1.60). The results of stratified analysis by age group showed similar results between two age groups, although the magnitude of odds ratios tended to be greater among older age group compared to younger age group.

**Table 3:** Multinomial regression for educational level on physical functional limitation level and stratified by age group.

	Age of education completed		
N	14 and younger 14,101	15 to 17 11,396	18 and older 3,637
**ALL**			
Needing assistant to carry out indoor activities			
n of cases	71	25	3
Adjusted OR (95% CI)	4.84 (3.61, 6.50)	2.21 (1.00, 4.86)	1.00
			
Needing assistant when going out			
n of cases	231	76	21
Adjusted OR (95% CI)	2.35 (2.03, 2.72)	1.08 (0.73, 1.60)	1.00
			
			
**AGE 50–59**	5,714	6,539	2,348
Needing assistant to carry out indoor activities			
n of cases	21	7	1
Adjusted OR (95% CI)	3.65 (1.67, 7.99)	1.68 (0.44, 6.46)	1.00
			
Needing assistant when going out			
n of cases	58	18	11
Adjusted OR (95% CI)	1.52 (0.14, 16.3)	0.39 (0.07, 2.27)	1.00
			
**AGE 60–69**	8,387	4,857	1,289
Needing assistant to carry out indoor activities			
n of cases	50	18	2
Adjusted OR (95% CI)	5.40 (3.00, 9.74)	2.48 (0.84, 7.29)	1.00
			
Needing assistant when going out			
n of cases	173	58	10
Adjusted OR (95% CI)	2.39 (0.29, 20.0)	1.33 (0.27, 6.52)	1.00

Adjusted OR = age and gender adjusted odds ratios

Table 4 shows that adjusted odds ratios of having physical functional limitations stratified by history of stroke. The odds ratios of having physical functional limitations for low education group relative to high education group were 2.07 (95%CI: 1.54, 2.80) for men and women without history of stroke and 1.85 (95%CI: 1.19, 2.89) for those with history of stroke. Further stratified analysis by gender showed that the respective odds ratios were 1.84 (95%CI: 1.19,2.88) and 2.20 (95%CI: 1.28, 3.80) among men, and 2.32 (95%CI: 1.53, 3.53) and 1.27 (95%CI: 0.59, 2.73) among women.

**Table 4:** Adjusted odds ratios of having physical functional limitations from logistic regression models

	ALL		MEN		WOMEN	
			
Age of education completed	14 and younger 14,101	15 and older 15,033	14 and younger 6,154	15 and older 7,624	14 and younger 7,947	15 and older 7,409
**History of stroke**						
NO (n)	13,624	14,659	5,873	7,364	7,751	7,295
n of cases	152	64	56	34	96	30
Age adjusted OR (95%CI)	2.07 (1.54, 2.80)	1.00	1.84 (1.19, 2.88)	1.00	2.32 (1.53, 3.53)	1.00
Multivariate OR (95%CI)	1.95 (1.44, 2.63)	1.00	1.66 (1.07, 2.84)	1.00	2.22 (1.46, 3.39)	1.00
						
YES (n)	477	374	281	260	196	114
n of cases	79	33	52	22	27	11
Age adjusted OR (95%CI)	1.85 (1.19, 2.89)	1.00	2.20 (1.28, 3.80)	1.00	1.27 (0.59, 2.73)	1.00
Multivariate OR (95%CI)	1.82 (1.17, 2.86)	1.00	2.05 (1.18, 3.56)	1.00	1.30 (0.60, 2.81)	1.00

Multivariate OR = age and marital status adjusted odds ratios.

## Discussion

This study showed that lower education level was associated with the higher prevalence of having physical functional limitation for both Japanese men and women which was consistent with the findings from previous studies in Europe and the United States [3,4]. In addition, the excess prevalence of having physical functional limitations was identified among men regardless of history of stroke, while the significant excess prevalence was observed among only women without history of stroke.

The associations between education level and physical functional limitations seem to be dichotomous rather than linear at least for the less severe functional limitation since our results did not show any significant difference in the odds of needing assistance when going outdoors between the high school graduates group and the higher education groups. On the other hand, we found relatively linear associations for the more severe physical functional limitation. This suggests that differences in physical functional limitations according to education level may be enhanced for more severe physical functional limitation. According to the results of stratified analysis by gender and age group, no age group difference was identified in the association between education level and functional limitations.

The present study identified that education level was inversely associated with prevalence of physical functional limitation among Japanese men and women. Although it was not our aim, we explored the possible mechanism for this phenomenon. The age and gender adjusted odds ratio for needing assistance to go out for the lowest educational group (2.04; 95% CI: 1.60, 2.60) was attenuated by adjustment for marital status, smoking behavior, drinking behavior, and medical history of stroke (1.77; 95% CI: 1.37, 2.28). This suggested that psychosocial and behavioral factors could have mediated some of the association between education level and functional limitations, although this interpretation was limited due to cross-sectional study design.

The stratified analysis by history of stroke showed that the unequal distribution of functional limitation according to education level was identified among Japanese community residents regardless of history of stroke. Lower education level associated with higher prevalence of having physical functional limitations among people without history of stoke may be explained by the unequal distribution of risk factors for physical functional limitations other than stroke, such as broken bones, dementia, and arthritic disorder [12]. Indeed, Matsuda et al[11] found an inverse association between education level and the prevalence of history of falling among Japanese elderly, while Liang et al[10] reported that less educated individuals were more likely to become cognitively impaired in a 3-year cohort study conducted in Japan. For unequal distribution of functional limitations among people with history of stroke, the differences in the severity of stroke [13,14], the level of functional recovery from stroke [14,15], the level of stroke care [10,20] depending on education level could be plausible reasons. Further studies are needed to examine possible explanations for the unequal prevalence of functional limitations by education level.

The results of further stratification by gender suggested gender differences in the prognosis of stroke between men and women. The excess prevalence of having functional limitation existed among men with low education level regardless of history of stroke. On the other hand, the excess prevalence of having functional limitation was identified only among women without history of stroke; smaller and not significant excess prevalence of functional limitation was identified among women with history of stroke.

Possible explanations for that gender difference could be due to differences in stroke subtypes between genders. In Japan, men had the higher proportion of ischemic stroke that often resulted in physical functional limitation more, compared to women [21]. That may be a reason for greater inequalities in physical functional limitations by education level among men than women.

Another explanation could be differences in social support from their spouse between genders. The proportion of men with history of stroke who had spouse were 79.2% for lowest education group, 88.3% for middle education group, and 94.3% for highest education group (p = 0.003), while women with history of stroke had 59.2%, 66.7%, and 69.0%, respectively (p = 0.40). This suggested that men with history of stroke have relatively higher proportion of having spouse and the proportion was more significantly different by education level compared to women. Previously conducted studies indicated that social support has been linked to improved functional recovery after stroke [22-24]. In addition, Ikeda et al. [25] showed that social support was associated with the higher risk of stroke mortality but not incidence for Japanese males and they suggested that social support may be a crucial factor for recovery from stroke especially for men. The proportion of having spouse, who was supposed to provide crucial social support, was different by education level among men with history of stroke and their prognosis from stroke may be influenced by social support more than among female counterparts. In the present study, additional adjustment by marital status attenuated but did not delete the excess prevalence of having physical functional limitation among men with low education attainment. Direct and reliable measure for social support could be necessary to examine the mediating effect of social support by spouse. Social support by friends and neighbors could also be crucial factors, in particular for women. However, unfortunately we did not have the data in the present study.

Moreover, education level may not be an equally-precise measure as an indicator of social stratification for men and women; household income or husband's educational level could be more appropriate indicator for social stratification for women [26]. It is also possible that small number of female cases may explain no significant association between education level and physical functional limitation.

The present study was one of the few studies examining the association between education level and physical functional limitation among Japanese men and women using a large group of Japanese community residents. Despites the strengths, our findings should be interpreted with several limitations in mind. Firstly, our study was limited to a single self-reported measurement of physical functional limitation. Secondly, our study was considered to be a cross-sectional study.

## Conclusion

In conclusion, the present study showed that lower educational attainment level was associated with higher prevalence of having physical functional limitations for both male and female Japanese community residents, and those without history of stroke. However, that association among persons with history of stroke was observed for men but not for women. This gender difference could be due to differences in stroke subtypes and social support.

## Abbreviations

(ORs): odds ratios; (95%CI): 95% Confidence interval.

## Competing interests

The authors declare that they have no competing interests.

## Authors' contributions

K.H. planned the present study, performed statistical analysis, and wrote the manuscript. H.I participated in its design and coordination of the cohort study and helped to plan the study and to write the manuscript. AI helped to plan statistical analysis. MI participated in its coordination of the cohort study and managed the data of the study. ST was planned and carried out the cohort study. All authors read and approved the final manuscript.

## Pre-publication history

The pre-publication history for this paper can be accessed here:


